# The Mechanical and Electrochemical Stability of Trimethysilane Plasma Nanocoatings Deposited onto Cobalt Chromium Cardiovascular Stents

**DOI:** 10.3390/ma17153699

**Published:** 2024-07-26

**Authors:** ThiThuHa Phan, John E. Jones, Yixuan Liao, Qingsong Yu, Meng Chen

**Affiliations:** 1 Department of Mechanical and Aerospace Engineering, University of Missouri, Columbia, MO 65211, USA; 2 Nanova, Inc., 1601 S Providence Rd, Columbia, MO 65211, USA

**Keywords:** plasma nanocoatings, cardiovascular stents, coating integrity & durability, cyclic polarization, corrosion resistance, hydrophilicity, hydrophobicity

## Abstract

The objective of this study was to evaluate the coating integrity performance and corrosion protection property of trimethylsilane (TMS) plasma nanocoatings that were directly deposited onto cobalt chromium (CoCr) L605 cardiovascular stents. Hydrophilic surfaces were achieved for the TMS plasma nanocoatings that were deposited onto the coronary stents through NH_3_/O_2_ (2:1 molar ratio) plasma post-treatment. With a coating thickness of approximately 20–25 nm, the TMS plasma nanocoatings were highly durable and able to resist delamination and cracking from crimping and expansion by a Model CX with a J-Crimp Station. The stent surface that was evaluated by Scanning Electron Microscopy (SEM) and Energy-Dispersive X-ray Spectroscopy (EDS) showed no indications of pitting, corrosion, or adsorption products on either the luminal or abluminal surfaces of the stents, in contrast to the uncoated stent surface. The TMS plasma nanocoatings significantly enhanced the stent’s corrosion resistance in immersion experiments that followed the ASTM F2129-15 corrosion protocol, evident in the increase of the open circuit potential (OCP) from 0.01 V for the uncoated L605 stent to 0.18 V for the plasma-nanocoated L605 stent, reducing potential cytotoxic metal ion release. Cyclic polarization (CP) curves show that the corrosion rate (density level) observed in plasma-nanocoated L605 stents was approximately half an order of magnitude lower than that of the uncoated stents, indicating improved corrosion protection of the stents. CP curves of the TMS plasma-nanocoated stents with different coating thicknesses show that, in the range of 20–65 nm, the coating thickness does not result in any difference in the corrosion resistance of the stents.

## 1. Introduction 

Since the first coronary stent implantation by Sigwart and Puel in 1986, coronary stent design continues to improve [1,2]. Early stent designs used thick struts to maintain patency while retaining the ability to visualize the stent during deployment via fluoroscopy [3,4,5,6,7]. However, the thicker struts were prone to thrombosis due to poor endothelialization and a large strut surface area [6]. Thinner strut stents (<100 µm) have since been developed that promote rapid endothelialization while maintaining adequate radial stiffness and strength [3,8,9]. Compared to the original stainless steel 316L stent platforms, newer alloys (i.e., MP35N, L605, and PtCr) have superior radiopacity, which assists with stent positioning during deployment [3]. Of even greater importance, the use of drug-eluting stents (DES) that are capable of gradually releasing cytostatic drugs that inhibit restenosis has revolutionized the field of interventional cardiology. 

Despite the improvements to the metal platforms, the evolution of thinner polymers X (used to hold drugs on the stent surface and gradually release them), and the use of newer cytostatic drugs, late stent thrombosis (defined as thrombosis occurring 1–12 months after percutaneous coronary intervention [PCI]) and very late stent thrombosis (thrombosis occurring over one year post-PCI) persist with DESs. Dual antiplatelet therapy (DAPT) is used to prevent stent thrombosis after PCI [10,11]. DAPT consists of a P2Y_12_ inhibitor in combination with aspirin to minimize platelet adhesion and activation [12,13]. However, DAPT significantly increases the bleeding risk. The patient-specific conditions that are associated with a high bleeding risk include an advanced age (>75 years), active cancer, a history of stroke, and the concomitant use of oral anticoagulant drugs [12,13,14,15,16,17].

Non-drug-eluting bioactive stent coatings have emerged as possible treatments for coronary artery blockages. Bioactive coatings both reduce in-stent restenosis and suppress late stent thrombosis. Hexacath markets a titanium-nitride-oxide-coated L605 stent that is superior to first-generation DESs and non-inferior to second-generation DESs [18,19,20,21]. The Hexacath stent delivery system has the CE mark and is marketed in Europe, but this stent system has not been approved by the US FDA [22]. Celonova has US FDA approval for Cobra, a stent consisting of a 50 nm layer of inorganic Polyzene-F (PzF) on a cobalt–chromium stent platform [23,24]. The PzF coating encourages healing and minimizes thrombosis [23,24]. Based on the SHIELD clinical trial, the PzF coated stent was found to be clinically effective for five years post-implantation [25,26]. The COBRA-REDUCE clinical trial further tested the feasibility of the discontinuation of DAPT 14 days after PCI with a Cobra stent. Unfortunately, the Cobra stent did not meet the clinical endpoint of reduced bleeding compared to patients with DESs receiving 3–6 months of DAPT [2,12]. However, the COBRA stent may be suitable in combination with single antiplatelet drug therapy, i.e., a P2Y_12_ inhibitor for one month post-implantation [27,28]. 

The TMS plasma coating process that was used in this study is based on a low-pressure glow discharge plasma process using trimethylsilane [(CH_3_)_3_-SiH] vapor as a precursor. The plasma that was generated using ammonia (NH_3_) and oxygen (O_2_) mixtures was used as a post-treatment to further modify the surface properties of the TMS plasma nanocoatings that were deposited onto CoCr L605 cardiovascular stents. The NH_3_+O_2_ plasma-treated TMS nanocoating (TMS+NH_3_/O_2_) suppressed smooth muscle cell proliferation while promoting endothelial cell migration in vitro. When applied to coronary stents, the nanocoating may be suitable for high-bleeding-risk patients who cannot tolerate DAPT for longer than one month. 

Our previous investigations [29,30] have demonstrated the biocompatibility of TMS+NH_3_/O_2_ plasma nanocoatings on L605 coupons for cardiovascular stent applications with promising efficacy in mitigating restenosis and thrombosis in coronary interventions. This study investigates the coating integrity performance and corrosion protection properties of TMS+NH_3_/O_2_ plasma nanocoatings that are deposited onto cobalt chromium (CoCr) L605 cardiovascular stents.

## 2. Materials and Methods

### 2.1. Sample Preparation 

CoCr L605 stents (1.3 mm in outside diameter × 12 mm in length, Resonetics Israel Ltd., Or Akiva, Israel) were cleaned using the process recommended by the manufacturer. Briefly, the stents were held in a sample rack that was immersed in a beaker containing ethanol for 15 min. The beaker containing the stents was then transferred to an ultrasonic bath held at 50 °C and sonicated for 30 min. The stents were removed from the bath, rinsed for 3 s with ethanol, and air-dried for 15 min. 

### 2.2. NH_3_/O_2_ Modified TMS Plasma-Nanocoated Stent

The detailed steps for plasma-nanocoating preparation have been described in our previous work [29]. TMS with a purity exceeding 97% was obtained from Gelest, Inc. (Morrisville, PA, USA). Anhydrous ammonia, with a purity surpassing 99.99%, was procured from Airgas (Holts Summit, MO, USA), and oxygen was sourced from Praxair (Columbia, MO, USA). The plasma deposition and modification system utilized an 80 L bell-jar reactor.

The plasma depositions and modifications employed a direct current (DC) power source. The titanium cathode was positioned between two grounded titanium anodes. The gas flow, pressure control, and nanocoating thickness were regulated for the oxygen pretreatment and TMS deposition processes as described [29]. The TMS deposition times were adjusted to determine the effect of thickness on nanocoating cracking and delamination (Section 2.4). With TMS deposition times of 10 s, 20 s, 30 s, and 40 s, the plasma coating thickness on L605 stents was 20–25 nm, 40–45 nm, 60–65 nm, and 75–80 nm, respectively. Coating thicknesses were measured by using a microscope-mounted, thin-film measurement device (Filmetrics F40-UV, KLA Corporation, Milpitas, CA, USA) [29].

Following the TMS nanocoating deposition, the reactor was evacuated to a base pressure of 1 mTorr, followed by the addition of an anhydrous ammonia–oxygen mixture to the reactor. The gas flow rates were 2.00 standard cubic centimeters per minute (sccm) and 1.00 sccm for ammonia and oxygen, respectively. When the pressure reached 50 mTorr inside the reactor, the plasma was sustained for 2 min at 5 W DC. To examine the effects of different NH_3_/O_2_ plasma post-treatment formulations, NH_3_:O_2_ gas ratios of 0:1, 1:0, and 2:1 were used for plasma post-treatment of the TMS plasma nanocoatings with sample designations as S_01_, S_10_, and S_21_, respectively.

### 2.3. Qualitative Coating Wettability Assessment

Deionized (DI) water was dropped onto the stent struts by using a micro-syringe attached to a contact angle meter/Goniometer—DMe 210 (Kyowa Interface Science Co., Ltd., Eden Prairie, MN, USA) with FAMAS 1.0 software. At least 4 droplets (0.5–0.7 μL) were tested on different parts of each stent for three stents (*n* = 3). Contact angle measurement on the stent struts was beyond the limitation of this equipment, but the shape of the droplets on each stent tells their surface hydrophilicity and hydrophobicity. The stents were kept in clean and dry conditions under room-temperature air for 2 years. The surface wettability of those aged stents was examined with water contact angle measurements over the time period of 2 years. 

### 2.4. Stent Dilatation Test with Different Coating Thicknesses

The adhesion of the plasma nanocoatings to the CoCr L605 stents was assessed after crimping and expanding processes that mimic stent dilatation procedures in clinical practice. Typically, a stent was positioned on a balloon catheter and crimped by a Model CX with a J-Crimp Station (Blockwise Engineering LLC, Tempe, AZ, USA) to a final crimping diameter of 0.8 mm. The crimped stent was expanded at 12 atm to reach a diameter of 3.2 mm. The stent was then examined under a Scanning Electron Microscope (SEM) (Quanta 600 FEG equipped with a Schottky Field Emitter (FEI Company, Hillsboro, OR, USA)) for coating integrity. An accelerating voltage of 10 kV with probe currents of 4.5 μA was used for the SEM operation.

The adhesion of the coating was also evaluated using a tracking fixture, following the methodology outlined in Standard F2394-07 [31]. The tracking fixture was immersed in a water bath that was maintained at a temperature of 37 °C ± 2 °C for a duration of 30 min. A stent was crimped onto a guide catheter with a guide wire inserted. Subsequently, the guide catheter was fully tracked into the fixture, utilizing a crosshead speed (pulling rate) of 2 inches per minute. The withdrawal process occurred at a comparable rate. To assess the stent’s trackability, the longest path was selected for testing. The nanocoating surfaces were then examined using SEM to identify any cracks, delamination, or other coating failures that may be present.

### 2.5. Immersion Test

The flowing model for immersion testing mainly includes a Masterflex peristaltic roller pump (Cole-Parmer Co., Vernon Hills, IL, USA), Masterflex Puri-Flex tubing (Cole-Parmer Co., IL, USA), and a water bath (Thermo Fisher Scientific, Waltham, MA, USA). Uncoated L605 and NH_3_/O_2_ modified plasma-nanocoated stents were inserted in series into tubing with an inner diameter of 3.1 mm. The plasma nanocoating thickness was about 15–20 nm. The tubing was placed within the head of the peristaltic roller pump. A simulated body fluid was prepared as described in [32]. The simulated body fluid (SBF) contained NaCl (8.035 g/L), NaHCO_3_ (0.355 g/L), KCl (0.225 g/L), K_2_HPO_4_·3H_2_O (0.231 g/L), MgCl_2_·6H_2_O (0.311 g/L), 1 M HCl (39 mL), CaCl_2_ (0.292 g/L), Na_2_SO_4_ (0.072 g/L), and Tris (6.118 g/L). About 7 mL of SBF was poured into the tube, with the two ends connected by an adapter, ensuring no air was inside the tubing. The temperature of the water bath was set at 37 °C and a pump speed of 36 rpm was performed for 7 days. After 7 days of circulation, the stents were removed, ultrasonically cleaned in ethanol for 15 min, then rinsed with DI water, and finally dried in room-temperature air. The morphologies of the stent surfaces were identified using SEM. Energy-Dispersive X-ray Spectroscopy (EDS) QUANTAX 200 (Bruker Axs, Madison, WI, USA) was used to determine the elemental components of any corrosion products. 

### 2.6. Electrochemical Test 

The electrochemical test procedure, as outlined in our previous publication [29], involved conducting cyclic polarization (CP) testing on both uncoated L605 stents and plasma-coated L605 stents (thickness approximately 15–20 nm) to evaluate their susceptibility to pitting. When designing the experiments, we followed the ASTM F2129-15 [33] corrosion protocol, which specifies a 1 h rest period for stabilization: “A 1 h rest period has historically been used to achieve relative stabilization”. Additionally, the CP scan was initiated when the rate of potential change was minimal (less than 3 mV/min). Following the ASTM F2129-15 corrosion protocol, each stent sample was secured with a stainless-steel wire, covered with epoxy, and placed in a corrosion cell containing phosphate buffered saline (PBS) at body temperature (37 °C) with stirring. After a 1.5 h open circuit measurement, CP testing was performed, and subsequent SEM was used to examine the signs of pitting corrosion. To analyze the impact of plasma nanocoating thickness on electrochemical behavior, three types of plasma-coated L605 coupon samples with coating thicknesses ranging from 20–25 nm, 40–45 nm, and 60–65 nm, respectively, were subjected to CP testing.

## 3. Results

### 3.1. Surface Wettability and Chemistry Assessment of Plasma-Coated Stents

**Figure 1:**
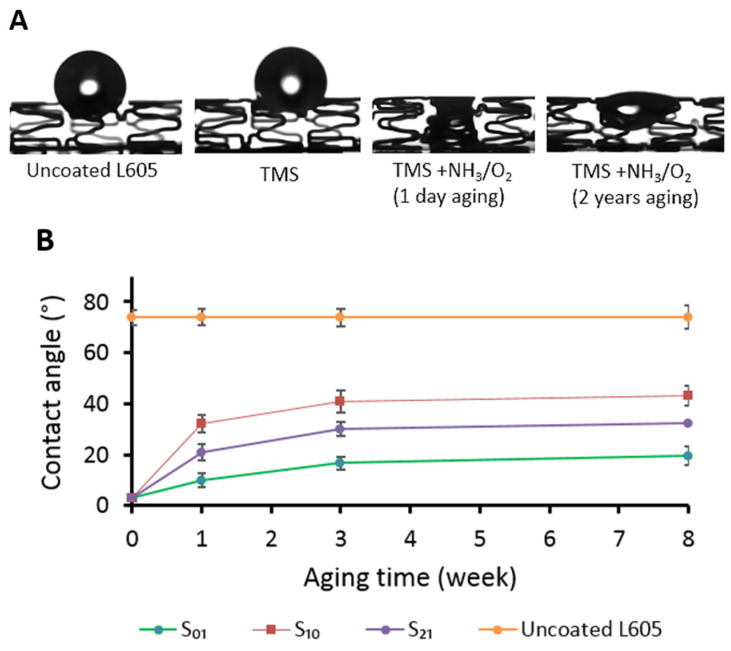
(**A**) Optical images of spherical water droplets on uncoated and plasma-nanocoated L605 stents. TMS+NH_3_/O_2_ nanocoatings are shown after 1-day and 2-year aging periods. (**B**) Surface static contact angles for uncoated and TMS+NH_3_/O_2_ plasma-nanocoated L605 coupons aged for up to 8 weeks, with six stents per group. S_01_, S_10_, and S_21_ samples were designated for NH_3_/O_2_ plasma post-treatment with NH_3_:O_2_ gas ratios of 0:1, 1:0, and 2:1, respectively.

TMS plasma nanocoatings with a thickness of 23.0 ± 1.4 nm were deposited onto CoCr stents with or without NH_3_/O_2_ plasma post-treatment. As illustrated in Figure 1A, the water contact angle measurement results indicate that these stents had different surface wettability compared to uncoated, bare stents. The uncoated stents and stents with TMS plasma nanocoatings but without NH_3_/O_2_ plasma post-treatment displayed hydrophobic surfaces. Consequently, water droplets were observed to remain in a spherical shape on the abluminal portion of the stent without spreading out on the stent surfaces. In contrast, with NH_3_/O_2_ plasma post-treatment, the plasma nanocoatings (TMS+NH_3_/O_2_) became hydrophilic. As a result, water droplets were able to spread along the stents’ struts and cover the luminal side. It is important to note that this hydrophilic effect persisted even after an aging period of 2 years after the deposition and modification of the coatings.

Figure 1B illustrates the results that were obtained from the contact angle measurements that were conducted on samples S_01_, S_10_, and S_21_, indicating a range between 20° and 40°. These values are lower than the contact angle that was measured on the uncoated L605 surface, which recorded a contact angle of 74°. It is evident that the TMS+NH_3_/O_2_ plasma post-treatment improved the surface wettability of the TMS plasma nanocoatings. The absence of the NH_3_ precursor in the plasma post-treatment (sample S_01_) renders the sample surface even more hydrophilic. On the other hand, the plasma post-treatment with NH_3_ plasma only (sample S_10_) was not as effective as that with O_2_ addition (samples S_01_ and S_21_) in improving the surface hydrophilicity.

### 3.2. TMS Plasma Nanocoating Integrity Following Crimping and Expansion 

Mechanical stability testing plays a crucial role in assessing coating defects, such as cracking or peeling, in stents. Coatings on stents are particularly vulnerable to cracking and delamination due to the substantial strains they experience during the crimping and expansion procedures involved in stent implantation [34]. These defects can lead to inflammation and unfavorable biological reactions. Given that stents are crimped onto balloon catheters for insertion into coronary arteries, it becomes imperative to evaluate the integrity performance of TMS+NH_3_/O_2_ plasma nanocoatings on stents following plastic compression and expansion.

**Figure 2:**
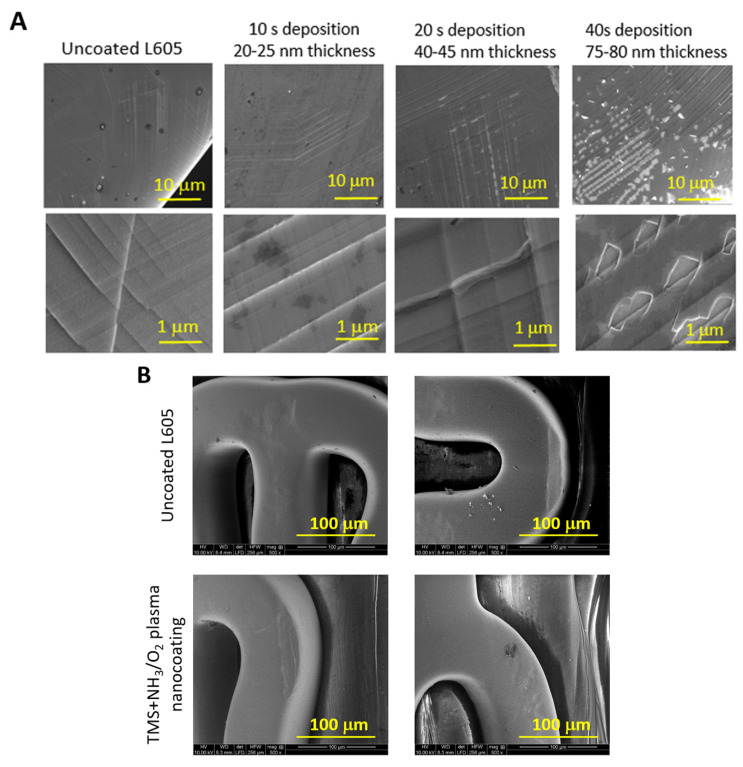
SEM images of (**A**) coating failures following the dilatation process on plasma-nanocoated L605 stents at different coating thicknesses; (**B**) stent surfaces after tracking fixture test, in which the coating thickness was 20–25 nm for TMS+NH_3_/O_2_ plasma nanocoatings.

SEM examination at high magnification was used to detect any potential coating failures on the stent surfaces, including both coating cracking and coating detachment. Figure 2 shows SEM images of both uncoated L605 stents and TMS+NH_3_/O_2_ plasma-nanocoated stents. These images were captured after undergoing the processes of stent crimping and expansion. At lower magnification, the plasma-nanocoated stent surfaces appear smoother and more conformal, without any discernible defects compared to uncoated stents. At higher magnification, however, numerous fold lines are visible vertically and horizontally on the stent surfaces, resulting from the crimping and expanding processes. The post-dilated surface texture of both uncoated stents and plasma-nanocoated stents reveals the presence of multiple shallow pits and ridges, a result of the dilation processes. It is worth noting that no micro-cracking or delamination was found in TMS+NH_3_/O_2_ plasma-nanocoated stents with a coating thickness of approximately 20–25 nm (Figure 2A). However, when the coating thickness increased to 40–45 nm, micro-cracks became apparent, and with even thicker plasma nanocoatings of 60–65 nm, clear instances of micro-delamination were observed. The severity of cracking and delamination increased with thicker coatings. Although the crack lengths and delamination sizes were around 1 μm, which is relatively small compared to the stent strut thickness of 80 μm [35], the impact of these minuscule failures should still be considered in biological assessments.

Before and after the tracking fixture test, the crimped stent remained securely positioned on the balloon catheter. Coating integrity is evaluated under demanding conditions, including bending, within the tracking fixture to simulate worst-case scenarios. The SEM images in Figure 2B demonstrate the intact stent surfaces, free from any signs of delamination or failures, even after undergoing crimping and delivery on the tracking fixture.

### 3.3. Corrosion Testing 

**Figure 3:**
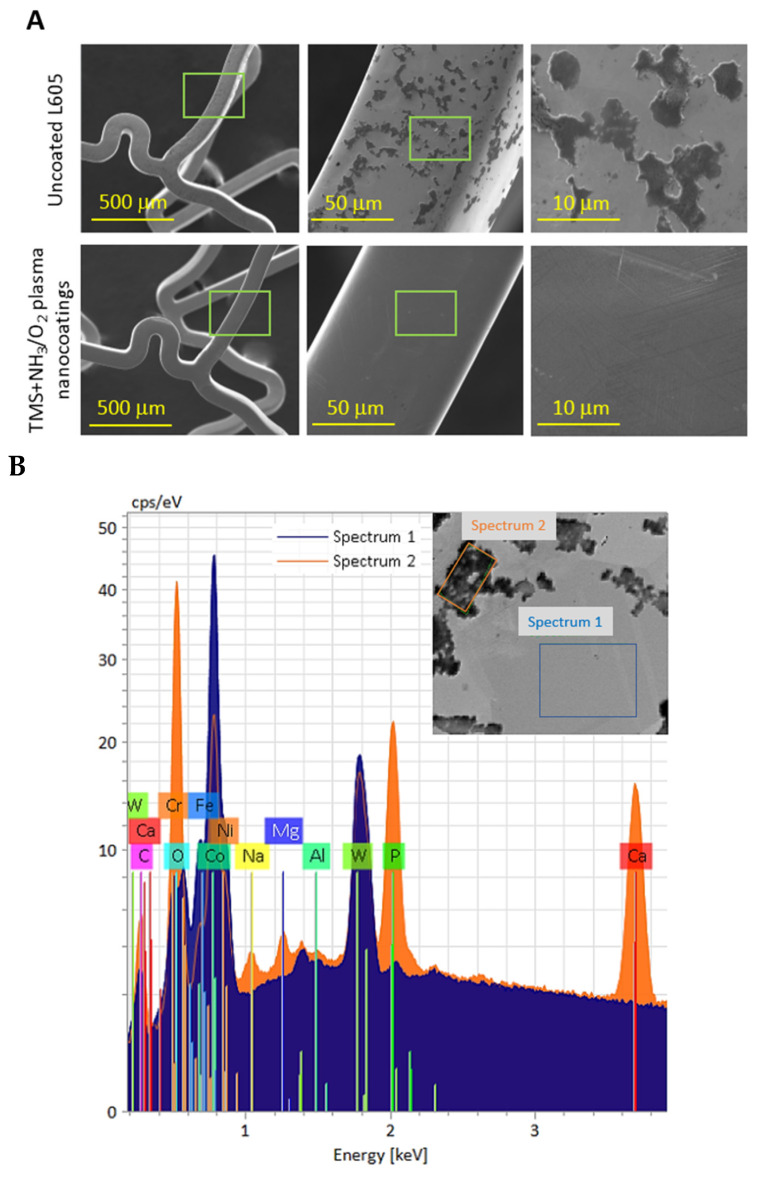
(**A**) SEM images for uncoated and TMS+NH_3_/O_2_ plasma-nanocoated L605 stents after 7-day immersion test in flow conditions; (**B**) EDS spectra of adsorbed mineral products on the stents. The TMS+NH_3_/O_2_ plasma nanocoating thickness was 20–25 nm.

The results of the immersion test conducted under flow conditions for the stents are presented in Figure 3. In the flow condition setup, uncoated L605 stents exhibited adsorption products primarily on the abluminal surfaces, whereas only a few corrosion products were observed on the luminal surfaces, which can be attributed to the circulation model used in the immersion test. In contrast, TMS+NH_3_/O_2_ plasma-nanocoated stents exhibited smooth and clean surfaces, showing no indications of pitting, corrosion, or adsorption products on either the luminal or abluminal surfaces.

Table 1 indicates the presence of mostly oxygen (O) (35.82 at%) and calcium (Ca) (11.6 at%), followed by phosphorus (P) (8.82 at%). This deposition is due to the diffusion mechanism of Ca and P from the solution near the interface between the alloy and the corrosion layer [36]. The presence of either P or Ca alone in TMS+NH_3_/O_2_ plasma nanocoatings can enhance the hemocompatibility, but not when acting together. Both Ca and P deposited in the coatings show inferior blood compatibility by the increase of adhered platelets [37,38]. On TMS+NH_3_/O_2_ plasma-nanocoated stent surfaces, the amount of O, P, and Ca is very low compared to that of uncoated stents. 

**Figure 4:**
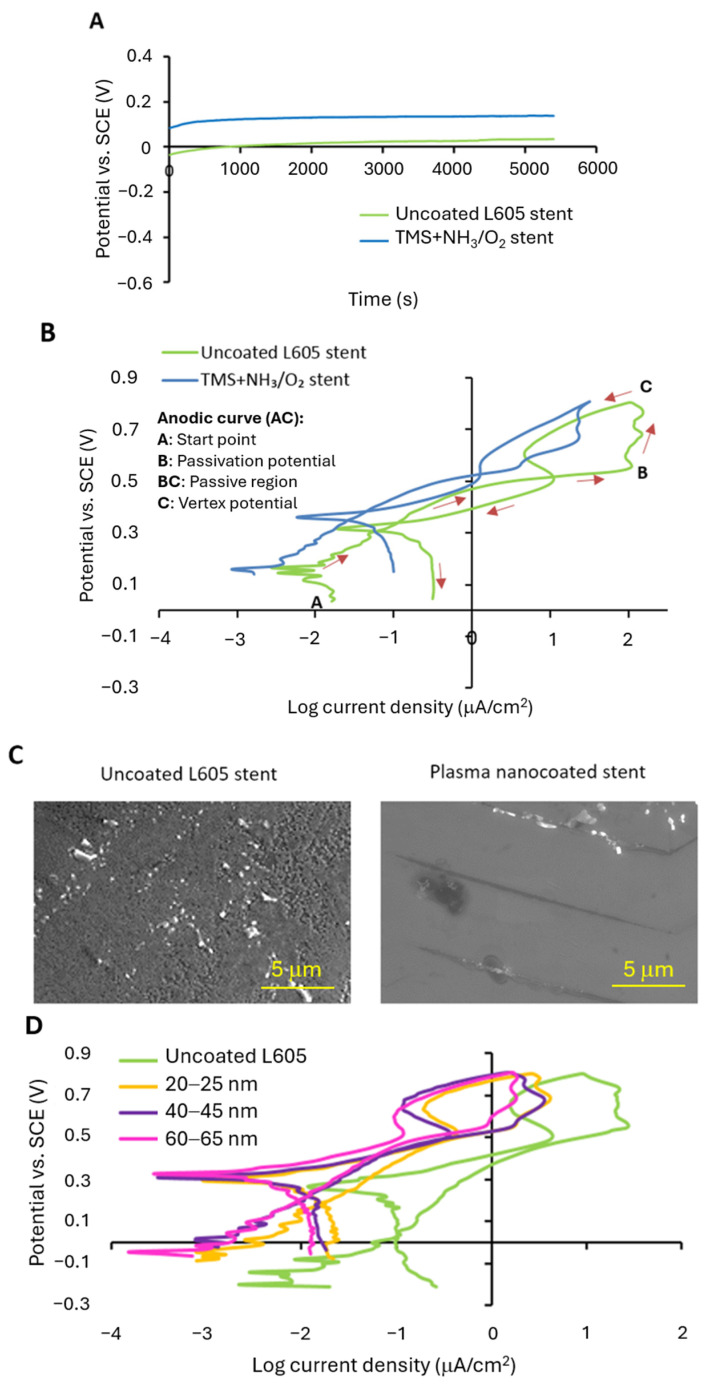
Electrochemical curves for uncoated and TMS+NH_3_/O_2_ plasma-nanocoated L605 stents and coupons: (**A**) Open circuit potential with immersion time; (**B**) Cyclic polarization (CP) curves; (**C**) SEM images for surface morphology of uncoated and plasma-nanocoated L605 stents after CP test; (**D**) CP curves of plasma-nanocoated coupons with different coating thicknesses.

Cyclic polarization (CP), an electrochemical method, was used to evaluate the corrosion properties of the uncoated and TMS+NH_3_/O_2_ plasma-nanocoated CoCr L605 stents. Before conducting the CP test, the open circuit potential (OCP) was monitored to clarify the sample surfaces’ stability in a particular corrosion environment. In OCP, time varies with changes in oxidation tendency of surfaces. The OCP displays the balance of surface oxidation and reduction. The oxidation and reduction processes involve the transfer of electrons, and the net sum of the electron transfer must be zero [39]. Therefore, at each value of the OCP, the total rate of oxidation is equal to the total rate of reduction. The OCP curves for the uncoated L605 and plasma-nanocoated stents are shown in Figure 4A. The OCP for the uncoated L605 stents in equilibrium is 0.01 V. When those uncoated L605 stents were treated with NH_3_/O_2_ plasma, the OCP increased to 0.18 V. The higher open circuit potential implies greater corrosion resistance. 

The CP curves for the uncoated and TMS+NH_3_/O_2_ plasma-nanocoated L605 stents are depicted in Figure 4B. These CP curves detect the relative susceptibility to localized pitting or crevice corrosion [39]. There are negative hysteresis loops in the CP curves of both the uncoated L605 stents and plasma-nanocoated stents. The occurrence of a negative hysteresis loop arises from a situation where the level of surface passivation is higher at more noble potentials. As a result, the current densities during the reverse scan are lower than the current densities at the same potential during the forward scan [40]. The implication is that the stent surfaces demonstrate effective surface passivation at more noble potentials, specifically around 0.5 to 0.8 V (vs. SCE). Consequently, the localized corrosion is prevented and the passivation film remains undamaged, leading to no pitting corrosion on stent surfaces. The corrosion rate (density level) observed in the plasma-nanocoated stents was approximately half an order of magnitude lower than that of the uncoated L605 stents. This suggests that the surfaces of TMS+NH_3_/O_2_ plasma-nanocoated stents exhibit enhanced corrosion resistance when compared to uncoated L605 stents. Figure 4C provides evidence supporting the resistance of the stent surfaces to pitting corrosion, as observed in the CP curves. The SEM images of the stent surfaces that were subjected to the CP test demonstrate the absence of pitting corrosion on the stent surfaces. In Figure 4D, the influence of the nanocoating thicknesses ranging between 20–25 nm, 40–45 nm, and 60–65 nm does not exhibit a discernible effect on the CP curves. All of the TMS+NH_3_/O_2_ plasma-nanocoated L605 coupon samples demonstrate CP curves that are similar to the uncoated L605 sample. However, resembling the stent CP curves, the corrosion rate observed on TMS+NH_3_/O_2_ plasma-nanocoated L605 coupons is approximately one order of magnitude lower than that of the uncoated L605 sample. Consequently, all TMS+NH_3_/O_2_ plasma-nanocoated L605 coupons, regardless of their coating thickness differences, exhibited superior corrosion resistance compared to their uncoated L605 counterparts.

## 4. Discussion

Thrombosis persists with current DESs, necessitating DAPT. Unfortunately, DAPT creates a risk for bleeding, particularly in high-bleeding-risk patients. Clinical investigations are currently focusing on reducing DAPT duration with DESs [12,41]. Non-drug-eluting bioactive stent coatings are being marketed with a target of reducing DAPT duration. However, while these stents may be competitive with drug-eluting stents with one-month DAPT, it is questionable that current bioactive stents can reduce DAPT duration to less than one month [12,28]. Short-duration DAPT of one month is desirable for patients with a high bleeding risk [42,43].

This study evaluated the mechanical and electrochemical stability of TMS+NH_3_/O_2_ plasma nanocoatings that were directly deposited onto CoCr L605 stents. Corrosion has been suggested as contributing to thrombosis and restenosis in the coronary vasculature. In this study, a 20–25 nm TMS+NH_3_/O_2_ plasma nanocoating remained firmly adhered to the stent strut with no indications of delamination following crimping, tracking, and expansion. The TMS+NH_3_/O_2_ plasma-nanocoated stents in this study exhibited enhanced corrosion resistance to simulated body fluid. Cobalt–chromium alloys are prone to oxidation, forming an oxide-rich surface passivation layer. The oxide layer is susceptible to degradation from body fluid [44,45,46]. With respect to stents, corrosion may be further exacerbated with overlapping stents across longer coronary lesions, resulting in accelerated damage to the passivation layer [47,48]. In this study, the TMS+NH_3_/O_2_ plasma nanocoating stabilized the surface, minimizing corrosion and particle release. We previously reported that the stent nanocoating suppressed Co, Cr, and Ni ion release from the nanocoated stents [29]. Corrosion particles can elicit an inflammatory response from macrophages and neutrophils [49,50]. Due to the more electrochemically noble surface imparted by the nanocoating (Figure 4), plasma-nanocoated stents were less susceptible to corrosion. The CP curves of the uncoated and plasma-nanocoated L605 stents are similar to reported curves, such as Ti-O modified stents [51], 316L stainless steel and Nitinol stent wires [39], and CoCrMo stents [52]. Pitting corrosion was not observed on the stent surfaces because, as the CP curves showed, at the pitting potential the current density started decreasing with the increase of potentials, implying that no pitting corrosion occurred. Pitting corrosion still exists on Ti-O modified stents as the existence of hysteresis loops on their CP curves show. Similar to CoCrMo alloy stents, plasma-nanocoated stents show a wide passive potential range (0.2 V_SCE_ to 0.55 V_SCE_), with low corrosion current density values (in the order of 10^−3^ μA/cm^2^). No pitting or crevice corrosion was observed with either CoCrMo stents or plasma-nanocoated stents. Similar to the CP curves of Ti-O modifed stents and 316L stainless steel stents, some peaks are observed at passive region. 

For plasma nanocoatings ranging from 20 to 65 nm, the polarization curves are similar. The similarity of the CP curves can be attributed to the small thickness difference from 20 to 65 nm and the presence of Si–O bonding in the plasma nanocoatings, which provides good corrosion resistance due to the lower dissolution rate of Si–O bonds compared to other metal oxides [29]. Figure 4D, however, does shows a slight decreasing trend in corrosion current with the coating thickness increase from 20–25 nm to 40–45 nm and 60–65 nm. The electrochemically passive nanocoating may reduce metal ion leakage to the coronary vasculature.

In this study, the electrochemical measurements were performed following ASTM F2129-15, in which a 1 h rest period was suggested. Before performing the CP test, as described in Section 2.6, a 1.5 h period was allowed for the stent specimens to stabilize to some degree in the test solution. As shown in Figure 4B,D, however, multiple exchanges in current densities (or multiple peaks) were observed with the polarization curves of both uncoated and coated stents. Such features generally indicate that the OCP was not attained. In other words, the OCP measurement duration of 1.5 h is insufficient for the purpose of testing the material’s corrosion. It should also be noted that the stents have a complex physical structure with cutting edges, contributing to localized high current densities and requiring a long time period to achieve stabilization. For future electrochemical characterization of stents, therefore, several hours to several weeks (or an even longer period) for the OCP measurement will be needed to evaluate the surface resilience against corrosion, especially for implants intended for human use. 

The TMS+NH_3_/O_2_ nanocoatings promoted endothelial cell proliferation while suppressing smooth muscle cell attachment and proliferation [29,30]. The cell behavior appears to be independent of the surface wettability of the nanocoating. The nanocoating with the 2:1 NH_3_+O_2_ surface treatment has intermediate wettability, between pure O_2_ and pure NH_3_ surface plasma treatments (Figure 1). Chen described NH_3_ and O_2_ plasma treatments for improving the vascular response of polytetrafluoroethylene (PTFE) vascular grafts [51]. A mixture of NH_3_+O_2_ for plasma surface modification of PTFE caused higher levels of endothelial cell attachment compared to either NH_3_ or O_2_ plasma alone. It was noted that water contact angle measurements were similar among the NH_3_, O_2_, and NH_3_+O_2_ groups. The NH_3_+O_2_ plasma treatments could lower platelet and leukocyte attachment to modified 316L stainless steel. The NH_3_+O_2_ treatment also outperformed either NH_3_ or O_2_ plasma alone in terms of reduced platelet and leukocyte attachment [53,54]. Our previous in vitro testing with the stent nanocoating found inhibited porcine coronary artery smooth muscle cell proliferation with nanocoated L605 coupons [29,30]. 

## 5. Conclusions

In this study, TMS+NH_3_/O_2_ plasma nanocoatings were successfully deposited onto coronary stents using a glow discharge plasma. The plasma nanocoatings exhibited robust adherence to the stent surface by remaining intact and maintaining resistance against deformation during crimping and expansion. Water contact angle measurements showed that the hydrophobic recovery of the plasma-nanocoated L605 stent surfaces mainly occurred in the first 3 weeks, and then the surface wettability stayed unchanged over the 2-year aging period. Notably, the coating enhances the stent’s corrosion resistance, increasing the open circuit potential to 0.18 V and reduchatting the corrosion rate by approximately half an order of magnitude, thereby minimizing potential metal ion leakage into body fluid. Because the nanocoatings promoted endothelial cell proliferation while suppressing smooth muscle cell attachment and proliferation, TMS+NH_3_/O_2_ plasma-nanocoated stents could potentially serve as an alternative for high-bleeding-risk patients, offering an alternative to drug-eluting stents with traditional dual antiplatelet therapy (DAPT).

## Figures and Tables

**Table 1:** Elemental compositions (O, P, and Ca) determined by EDS for TMS+NH_3_/O_2_ plasma-nanocoated stent surfaces (at%) and uncoated L605 stents (at%), (*n* = 3).

Elements	TMS+NH_3_/O_2_ Plasma-Nanocoated Stents (at%)	Uncoated L605 Stents (at%)
O	2.10 ± 0.3	35.82 ± 2.32
Ca	0.01 ± 0	11.6 ± 2.01
P	0.07 ± 0.01	8.82 ± 0.56

## Data Availability

The original contributions presented in the study are included in the article, further inquiries can be directed to the corresponding author.
